# Mind over matter

**DOI:** 10.4103/0972-0707.45245

**Published:** 2008

**Authors:** L Lakshmi Narayanan

**Affiliations:** Hon. Gen. Secretary, F.O.D.I, Principal, SRM University Dental college, Chennai

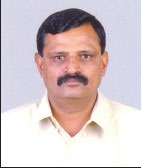



Having been in this profession for close to three decades, the last twenty six years as a teacher, I would like to reflect on the changes I have observed, share some of my thoughts, and ask a few questions. “There is nothing permanent except change” is an often quoted adage. Are all changes for the good is the moot question. Changes like verifiable credit points for continuing to practice is most welcome. But do all changes fit this category? A lot has been done to stem the rot. The mindset of the teachers and students has undergone a tremendous shift – not necessarily for the good. “Teachers working full time” in institutions a thousand miles away – is this change welcome? It is said by some that the present day students care less for their teachers. If true, who is responsible? Are the present day younger teachers as dedicated as retired teachers or the seniors of today? Is money becoming the top priority? In India, can we really expect a move in the right direction? To borrow some words from the US President elect Barrack Obama – Yes, we can. I sign off with the quote from the Mahatma “Be the change you want to see.”

